# Supplementary material for: Polyphenol Profile, Antioxidant Activity, and Hypolipidemic Effect of Longan Byproducts
Source: Molecules. 2023 Feb 23;28(5):2083. doi: 10.3390/molecules28052083 (PMC10004001; doi:10.3390/molecules28052083)
Supplement: Supplementary file 1 [file molecules-28-02083-s001.zip › molecules-2184911-supplementary.pdf]

Raw western images

Figure S1: The original western blot images.

(1) PPARα

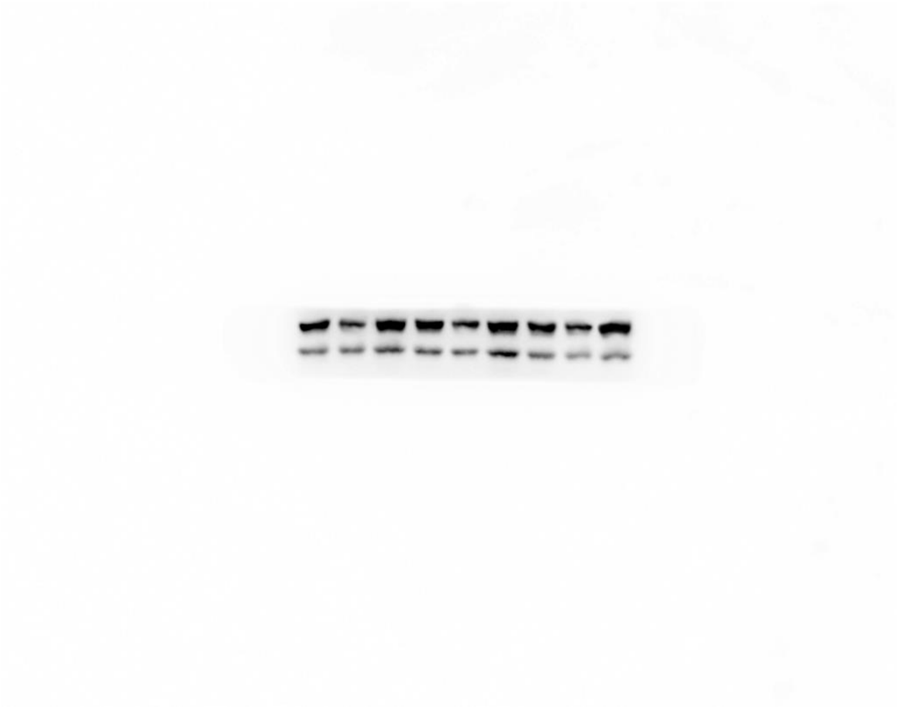

(2) LXRα

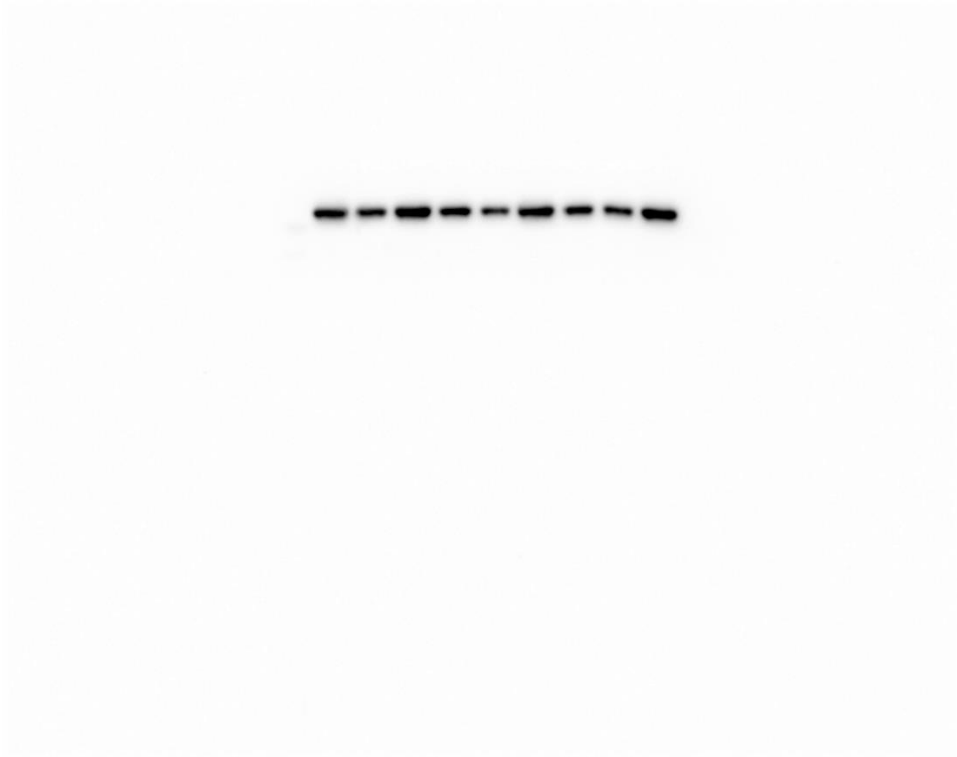

(3) FAS

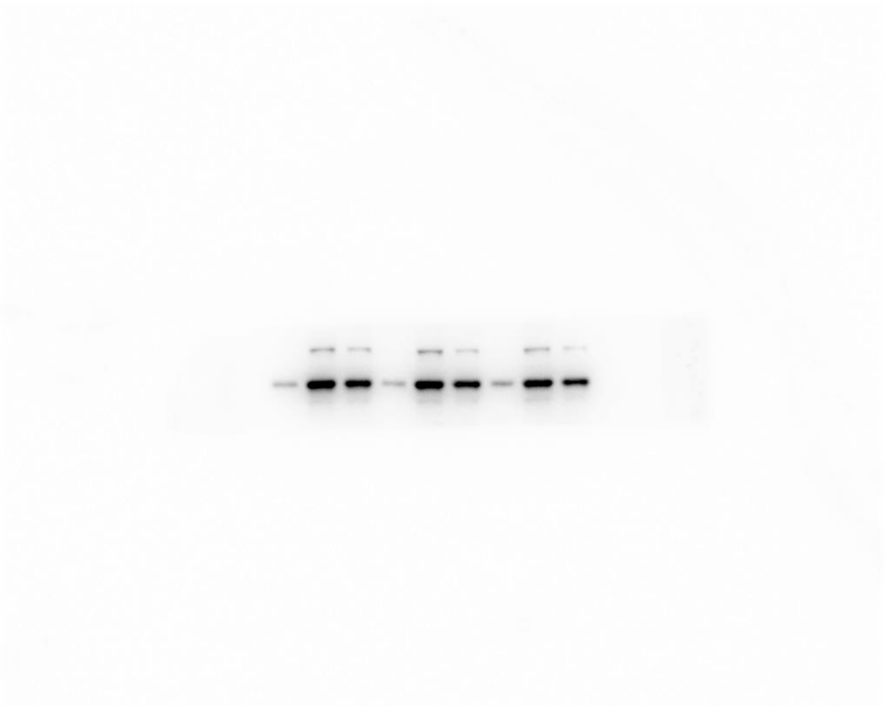

(4) CYP7A1

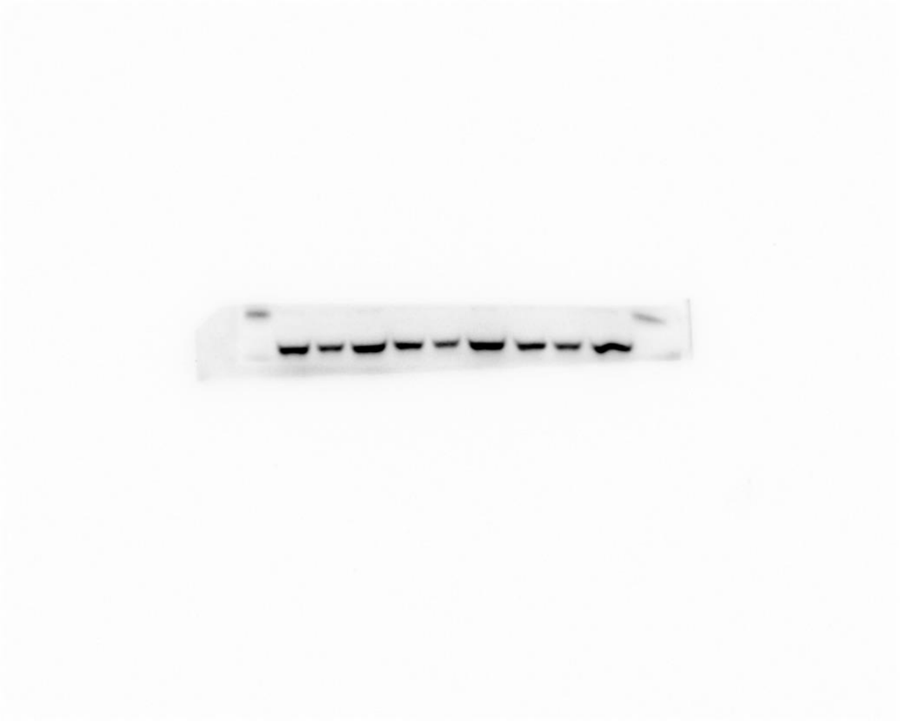

(5) β-actin

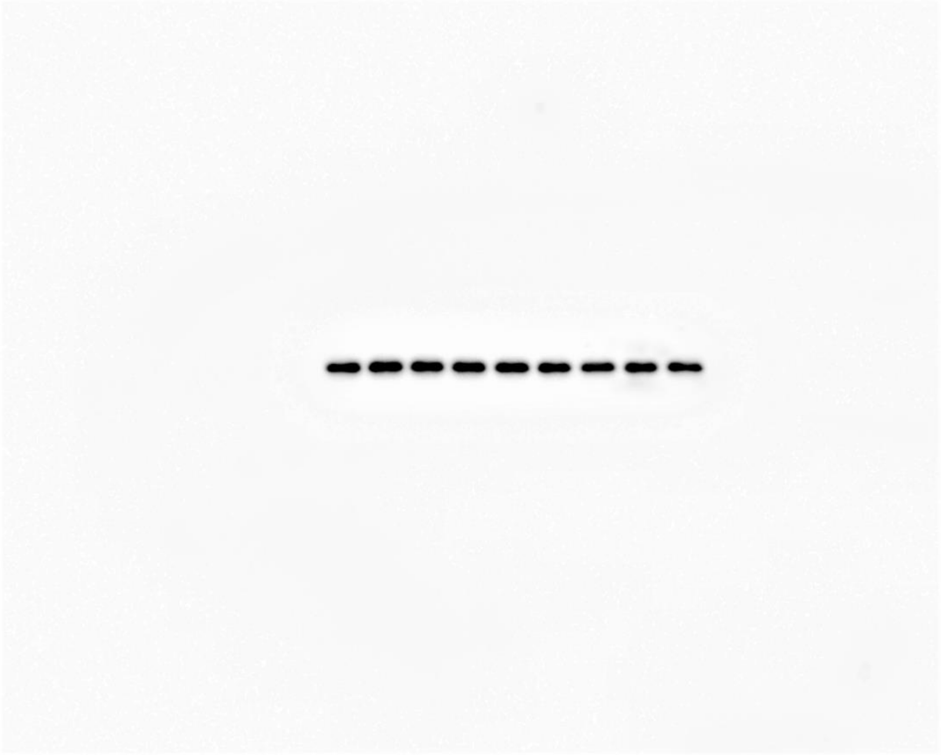

Table S1. Densitometry Readings for all the bands.

Lane	1	2	3	4	5	6	7	8	9
**PPARα**	3072	930	4721	3536	1182	4991	2983	1374	5177
**LXRα**	2391	563	5029	2156	632	5204	2259	639	5261
**FAS**	871	4473	3141	971	4393	3209	1162	4015	2970
**CYP7A1**	5008	1839	6321	4567	2267	6830	3634	1769	5475
**β-actin**	4136	4485	4261	4254	4220	3999	3878	4052	3949
